# Microglia remodeling in the visual thalamus of the DBA/2J mouse model of glaucoma

**DOI:** 10.1371/journal.pone.0323513

**Published:** 2025-05-15

**Authors:** Jennifer L. Thompson, Shaylah McCool, Jennie C. Smith, Victoria Schaal, Gurudutt Pendyala, Sowmya Yelamanchili, Matthew J. Van Hook

**Affiliations:** 1 Department of Ophthalmology & Visual Sciences, Truhlsen Eye Institute, University of Nebraska Medical Center, Omaha, Nebraska United States of America; 2 Department of Pharmacology & Experimental Neuroscience, University of Nebraska Medical Center, Omaha, Nebraska United States of America; 3 Department of Anesthesiology, University of Nebraska Medical Center, Omaha, Nebraska United States of America; 4 Department of Cellular & Integrative Physiology, University of Nebraska Medical Center, Omaha, Nebraska, United States of America; University College London, UNITED KINGDOM OF GREAT BRITAIN AND NORTHERN IRELAND

## Abstract

Microglia are the resident immune cells of the central nervous system and mediate a broad array of adaptations during disease, injury, and development. Typically, microglia morphology is understood to provide a window into their function and microglia have the capacity to adopt a broad spectrum of functional phenotypes characterized by numerous morphologies and gene expression profiles. Glaucoma, which leads to blindness from retinal ganglion cell (RGC) degeneration, is commonly associated with elevated intraocular pressure (IOP) and triggers microglia responses within the retinal layers, at the optic nerve head, and in retinal projection targets in the brain. The goal of this study was to determine the relationship of microglia morphology to intraocular pressure and the loss of RGC output synapses in the dorsolateral geniculate nucleus (dLGN), a RGC projection target in the thalamus that conveys information to the primary visual cortex. We accomplished this by analyzing microglia morphologies in dLGN sections from DBA/2J mice, which develop a form of inherited glaucoma, at 4, 9, and 12 months of age, representing distinct time points in disease progression. Microglia morphology was analyzed using skeletonized Iba1 fluorescence images and fractal analyses of individually reconstructed microglia cells. We found that microglia in older DBA/2J mice adopted simplified morphologies, characterized by fewer endpoints and less total process length per microglia cell. There was an age-dependent shift in microglia morphology in tissue from control mice (DBA/2J^Gpnmb+^) that was accelerated in DBA/2J mice. Measurements of microglia morphology correlated with cumulative IOP, immunofluorescence labeling for complement component C1q, and vGluT2-labeled RGC axon terminal density. Additionally, fractal analysis revealed a clear distinction between control and glaucomatous dLGN, with microglia from ocular hypertensive DBA/2J dLGN tissue showing an elongated rod-like morphology. RNA-sequencing of dLGN showed an upregulation of immune system-related genes. These results suggest that microglia in the dLGN alter their physiology to respond to RGC degeneration in glaucoma, potentially contributing to CNS adaptations to neurodegenerative vision loss.

## Introduction

Glaucoma is an age-related neurodegenerative disorder of the visual system and one of the leading causes of blindness and visual impairment worldwide [1–4]. All forms of glaucoma are characterized by optic nerve atrophy and irreversible vision loss due to the progressive death of retinal ganglion cells (RGCs), the only output neurons of the retina [5–7]. The two most prominent glaucoma risk factors are age and elevated intraocular pressure (IOP), and the cornerstone of glaucoma management is to lower IOP [5]. Chronic IOP elevation (ocular hypertension; OHT) creates mechanical stress at the optic nerve head (ONH), damaging the unmyelinated region of RGC axons as they converge to exit the eye through a meshwork of glial and structural support cells [8–10]. This triggers a variety of inflammatory responses which become pathological and contribute to RGC degeneration [11,12]. The degenerative events that follow occur in a slow and compartmentalized manner, giving rise to pathological asynchrony between RGC somas, dendrites, and axons, as well as spatiotemporal differences among neuron-glia dynamics, particularly those involving microglia.

Microglia are highly plastic sentinels of the central nervous system that preserve synaptic integrity throughout the brain by surveilling the parenchyma and facilitating rapid, specialized immune responses to a variety of intra- and extracellular threats, including IOP-induced RGC stress [13–15]. Microglia also maintain homeostasis within local circuits by making direct, repeated contact with nearby synapses to counteract perturbations in neuronal health and transmission [13]. The responses of retinal microglia to aging and IOP elevation are well-documented. Aging leads to decreased phagocytic capacity, increased cytokine production, and slower migration to sites of injury [16,17], while sustained IOP elevation causes retinal microglia to retract their processes and adopt pro-inflammatory phenotypes. Specifically, OHT causes retinal microglia to release IL-1α, TNFα, and C1q (interleukin-1 alpha, tumor necrosis factor alpha, and complement component 1q) —proinflammatory mediators that drive neurotoxic astrogliosis [18–20]. In support of these findings, a systematic review recently concluded that ameboid microglia, reactive astrocytes, and proinflammatory cytokines frequently aggregate near the ONH in glaucoma donor tissues [21].

Despite the relevance of retinal gliosis, the way that neuron-glia interactions within retinorecipient brain regions respond to glaucoma risk factors is less studied. Immune interactions at these distal sites are likely to be equally important in the preservation and health of RGCs. For instance, evidence from experimental glaucoma models suggests that many of the earliest neurodegenerative changes in glaucoma impact visual centers of the brain [22–28]. IOP elevation drives early remodeling in local circuits within the visual thalamus (dorsolateral geniculate nucleus; dLGN), which is the retinorecipient nucleus required for conscious vision [22,27,29,30]. In the superior colliculus, a more distal vision center, RGC axon terminals remain intact but show early signs of degeneration, including mitochondrial abnormalities and bouton atrophy [31]. At later stages in humans, transneuronal degeneration becomes widespread and glaucoma-specific circuit impairment affects multiple white matter tracts related to visual processing, and neuronal loss causes gray matter reductions within the visual nuclei and cortices of advanced glaucoma patients [10,32–37].

Our primary objective in the current study was to determine how aging and OHT impact the integrity of retinogeniculate synapses and the state of dLGN microglia during glaucoma progression. We address this using DBA/2J mice, a widely used and extensively characterized mouse model of glaucoma that develops age-related OHT due to mutations in two genes: glycosylated protein nmb (*Gpnmb*) and tyrosinase-related protein 1 (*Tyrp1*) [38–41]. Although comparable mutations are not associated with glaucoma in human patients, the DBA/2J mice exhibit IOP-related visual system pathology akin to human glaucoma, making them a useful rodent glaucoma model [38,41–43]. Here, we found that dLGN microglia in older DBA/2J mice become less morphologically complex and adopt a bipolar or “rod-like” morphology [44–47] in a manner that correlated with IOP and loss of immunofluorescence staining for retinal ganglion cell axon terminals. We also found that microglia morphology changes correlated to increased labeling of complement C1q in the dLGN, potentially involved in targeting degenerating RGC axon terminals and other debris for microglial phagocytosis [13,48,49]. Finally, bulk RNA sequencing of dLGN pointed toward increased expression of genes associated with immune responses.

## Materials and methods

### Animals and tissue processing

All protocols involving animals were approved by the Institutional Animal Care and Use Committee at the University of Nebraska Medical Center. Male and female DBA/2J (Jackson Labs #000671, RRID:IMSR_JAX:000671) and DBA/2J^Gpnmb+^ (Jackson Labs #007048, RRID:IMSR_JAX:007048) mice were bred onsite and housed socially under a 12/12-hour light/dark cycle with free access to food and water*.* Eye pressures (left and right) were measured (in mmHg; millimeters of mercury) from the central cornea using a handheld rebound tonometer (TonoLab, iCare) calibrated for mouse eyes. Measurements were obtained while mice were under light anesthesia (2% isoflurane) within five minutes of losing their righting reflex. Baseline IOPs were recorded when mice were approximately 3 months old, then IOPs were measured monthly until sacrifice, with all subsequent recordings performed at or near the same time of day to minimize the effects of diurnal variance. Each IOP reflects the average of three tonometer readouts, which each comprise six consecutive, low-error measurements; thus, a total of 18 measurements were obtained per eye per session. At 4-, 9-, and 12-months (mo) of age, mice of each strain were euthanized via CO_2_-inhalation followed by cervical dislocation. Immediately afterwards, brains were dissected, rinsed, and drop-fixed in 4% paraformaldehyde (PFA) in PBS (phosphate buffered saline) for 4 hr, shaking gently at room temperature. After 3x10 min washes, brains were cryoprotected in a 30% sucrose solution and stored at 4°C until fully saturated. Then they were embedded in tissue molds with 3% agar in PBS. Coronal sections (50 µm) containing the dLGN were obtained using a Leica VT1000S vibratome, mounted onto SuperFrost Plus slides, and stored at -20°C until use. No animals or tissues were excluded.

### Immunohistochemistry

Sections containing the widest region of the central dLGN were chosen for immunostaining. Slides were briefly rinsed in PBS, then blocked and permeabilized (1 h) at room temperature in a 7.4 pH buffer (5% donkey serum, 5% goat serum, 0.5% Triton X-100). Sections were co-immunolabeled with 200 µL of buffer diluting primary antibodies against vesicular glutamate transporter 2 (anti-vGluT2; Cat#6D2092; Millipore, RRID: AB_2665454) at 1:250, and ionized calcium binding adaptor protein 1 (anti-Iba1; Cat#019-19741, Wako; RRID: AB_8395054) at 1:500, to visualize RGC axon terminals and microglia, respectively. To enhance the resolution of fine microglial processes, primary antibodies were incubated for three overnights. A subset of DBA/2J (n = 32) and DBA/2J^Gpnmb+^ (n = 25) tissues were immunostained (one overnight) using a knockout-validated anti-C1q antibody (Cat#ab182451, Abcam; RRID: AB_2732849) at 1:500. A guinea pig polyclonal antibody against NeuN was used at 1:500 dilution to label dLGN neurons (Cat#ABN90, Millipore, RRID: AB_11205592). Unbound primary antibodies were cleared with 6x10 min washes in PBS, then sections underwent an additional block (1 h) prior to dark-incubation (3 h) with a 1:200 dilution of Alexa Fluor-conjugated secondary antibodies: donkey anti-rabbit IgG (H + L), Alexa Fluor 568-conjugated (Cat#A10042; RRID: AB_2534017) and/or goat anti-guinea pig IgG (H + L), Alexa Fluor 488-conjugated (Cat#A-11073; RRID: AB_2534117). Sections were washed (3x10min), briefly rinsed (dH_2_O), then coverslipped using VECTASHIELD HardSet mounting medium (Cat#H1400).

#### vGluT2 puncta analysis.

To visualize immunofluorescence, volumetric images (1 µm z-spacing) were acquired at high resolution (370 x 370 µm; 1024 x 1024 pixels) through central dLGN core. All z-stacks were pre-processed to average 4 slices per plane, then compressed stacks were trimmed to 40 µm (z-span). To visualize vGluT2+ puncta, a single high-intensity mid-section plane was isolated in ImageJ/Fiji and a median filter (*Despeckle*) was applied to enhance contrast. vGluT2 immunolabeled RGC axon terminals were detected using the *Synapse Counter* plug-in with the following settings: rolling ball radius, 10; max filtering, 2; thresholding, OTSU; size restrictions, 100–7500 pixels), then an RGB output was selected and quantified with *Analyze Particles* (size: 6–100 µm^2^; circularity: 0–1). Image acquisition area was incorporated into the count from the summary output to calculate puncta densities. To measure C1q intensity, backgrounds were subtracted throughout each stack using a rolling ball radius set to 50 pixels. The average pixel intensity of each plane was determined (*Measure*), then the frame with the highest mean intensity value was identified and its mean value was recorded.

#### NeuN cell density analysis.

A series of images (370x370 µm, 1024 x 1024 pixels) was acquired through the z-axis (1 µm spacing) from the central region of dLGN tissue sections of 12 mo DBA/2J and DBA/2J^Gpnmb+^ control mice. Four images were acquired per plane and averaged to reduce noise. NeuN+ neurons were manually counted in a three-dimensional volume (370x370x30 µm) using the Cell Counter plug-in in ImageJ and the resultant count was scaled to provide the number of NeuN+ cells per mm^3^. Display images are from maximum intensity projections.

#### Global skeleton analysis.

Microglia skeleton analysis was performed with a method adapted from Young and Morrison (2018) [50]. The Iba1+ channel from each uniformly-trimmed z-stack was projected onto a single plane based on maximum intensity values. In effect, this generates a two-dimensional surface that enhances signals arising from microglial fine processes but maintains their overall morphological integrity. After conversion to an 8-bit black and white image, edges were enhanced using an unsharp mask filter set to a 0.5 pixel sigma radius and a 0.6 mask weight. Impulse noises were removed using the *Despeckle* function before and after global thresholding (modified IsoData algorithm: 22%). The *Close* command was run to bridge gaps in adjacent microglial processes that had perhaps eroded or provided weak signal, then dark noise was smoothed by running the *Remove Outliers* command with a 7 pixel radius and a deviation threshold of 50 against the surrounding median. Running the *Skeleton (2D/3D)* command generated final skeletonized images that were subsequently analyzed using the *Analyze Skeletonize 2D/3D* plugin. From the output windows, endpoint and branch length data were saved and trimmed to exclude small (<1 µm) incomplete (<2 endpoints) branches. To calculate branching and endpoint averages per microglia across the full field of view, Iba1+ cells were counted through each 40 µm Z-stack using the *Cell Counter* plug-in.

#### Fractal analysis.

To analyze the morphology of individual microglia [50–52], 216 Iba1+ cells were hand-traced throughout the same set of 40 µm image stacks. One Iba1+ cell in the central region of each quadrant (4 per dLGN) was chosen on the basis of being fully contained within the image volume. A 3D Gaussian Blur filter was applied to the z-stack with a 1.0 sigma radius on each plane. In a duplicate stack, images were converted to masks with the “redirect to” option of the *Analyze Particles* function. Selected Iba1+ cells were traced through the masked volume using the *Blow and Lasso Segmentation Tool*, and regions of interest (ROIs) were frequently redirected to the unmasked, filtered z-stacks for reference. Final 2D outlines were added to the ROI Manager and saved in sets of four. A duplicate image was created for every microglial trace, in which the background had been cleared and the corresponding ROI was filled with the foreground color, resulting in a set of uniformly-sized 2D images each containing a single microglia silhouette. ROIs were outlined using the *Binary* options and whole images were analyzed using the *FracLac* plugin [52] set to box-counting method; 4 start positions; 45% largest grid size with *Hull & Circle* results selected. Thresholds for masking were determined on a sample-by-sample basis, and occasionally single pixels were manually inserted for the purpose of filling or recreating gaps (i.e., between pseudo-overlapping processes). All final outlines were meticulously retouched to align with the true, unmasked signal.

### RNA sequencing

Bulk RNA sequencing was performed with a total of eight samples from 9-month-old DBA/2J and DBA/2J^Gpnmb+^ control mice with each sample containing dLGN tissue pooled from three mice. To isolate dLGN tissue, brains were dissected into an RNAse-free slush of phosphate buffered saline and cut into 500 μm-thick sections on a vibratome. The dLGN from both hemispheres was then identified and dissected free. dLGN tissue from three mice was pooled into each sample and weighed before being flash frozen on dry ice. Total RNA was isolated from dLGN tissue using the Direct-Zol RNA kit (Zymo Research, Irvine, CA). Approximately 1 µg of RNA per sample was sent on dry ice to LC Sciences for RNA sequencing with their Poly(A) RNA sequencing service. The Poly(A) RNA sequencing library was prepared using the TruSeq-stranded-mRNA protocol (Illumina) and purified using oligo-(dT) magnetic beads. After purification and DNA library construction, quality control and quantification was performed using the 2100 Bioanalyzer High Sensitivity DNA Chip (Agilent Technologies) and paired-end sequencing performed using the NovaSeq 6000 sequencing system (Illumina). Cutadapt and Perl scripts were used by LC Sciences for bioinformatics analyses to remove reads containing adaptor contamination, low-quality bases, and undetermined bases and sequence quality assessed using FastQC. HISAT2 was used to map reads to the genome and reads of each sample were assembled using StringTie, after which transcriptomes were merged to reconstruct a comprehensive transcriptome. StringTie and Ballgown were then used to estimate the expression levels of all transcripts.

### Statistics

All statistical tests were performed in GraphPad Prism 10. To represent the cumulative effect of IOP across time, longitudinal IOP data were compressed into a single AUC integral (area under the curve in mmHg*days). Two-way analysis of variance (ANOVA), factoring strain and age, were used for group comparisons, followed by Šídák-corrected pairwise comparisons. Comparisons involving individual Iba1+ features were performed using nested one-way ANOVAs followed by Tukey’s HSD tests. To test explanatory relationships between variables, simple linear regressions were used and follow-up slope comparisons (two-sided) were performed. P-values < 0.05 were considered statistically significant. Data are reported as mean ± standard deviation in bar graphs and 95% confidence intervals in regression plots.

## Results

**Fig 1:**
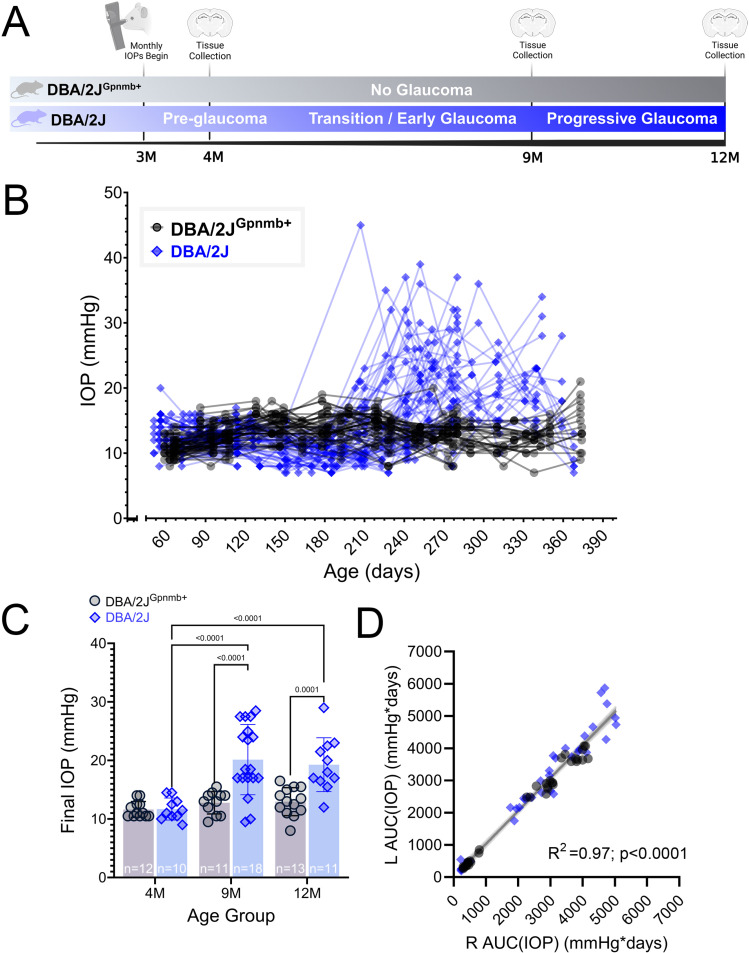
Intraocular pressure measurements in DBA/2J mice. **(A)** Experimental design indicating timing of IOP measurements and tissue collection. **(B)** IOP measured in individual eyes (left and right) from mice included in the present study. **(C)** Group data of final left eye IOP measurements taken before tissue collection. The p-values represent pairwise Tukey’s multiple comparison tests following two-way ANOVA. **(D)** Scatter plot and linear regression of integrated IOP [AUC(IOP)] from left and right eyes. Shaded region represents 95% confidence interval.

To determine the extent and time-course of OHT in our DBA/2J colony, we made monthly IOP measurements from the left and right eyes of 39 DBA/2J mice and 36 DBA/2J^Gpnmb+^ controls (Fig 1A and 1B). Although DBA/2J^Gpnmb+^ mice carry the *Tyrp1*^*b*^ allele they are spared from severe iris disease and do not become ocular hypertensive [39,40]. In line with this, DBA/2J^Gpnmb+^ IOPs were comparable across all ages (4mo-9mo: *p = 0.75*; 9mo-12 mo: *p = 0.99*; Tukey’s multiple comparisons), with IOP averages of 11.6 ± 1.4 mmHg at 4-months, 12.8 ± 1.9 mmHg at 9-months, and 13.0 ± 2.4 at 12-months (Fig 1C). In contrast, age-related IOP elevations were clearly apparent in DBA/2J eyes in both 9- and 12-month conditions (4mo-9mo: *p* < 0.0001; 4mo-12mo: *p* = 0.0001; Tukey’s multiple comparisons) (Fig 1C). DBA/2J IOPs were similar to age-matched controls at 4mo (*p = *0.96, Tukey’s multiple comparisons), and DBA/2J IOPs were significantly elevated in comparison to controls at 9mo and 12mo (9mo: 20.1 ± 6.0 mmHg, *p* < 0.0001; 12mo: 19.3 ± 4.6, *p = *0.0001; Tukey’s multiple comparisons) (Fig 1C). As a measure of sustained IOP elevation, we calculated the area under the curve (AUC) of the IOP series from individual mice, which generated AUC(IOP) values for each eye, in mmHg*days. Using these measurements, we found that left eye and right eye IOP measurements were tightly correlated with each other for individual mice [Fig 1D; F(1,73)=2043, R^2^ = 0.97, p < 0.0001]. These data verify that age-related OHT is conserved within our DBA/2J colony and demonstrate that our age groups are appropriate for comparisons of prolonged IOP exposure.

**Fig 2:**
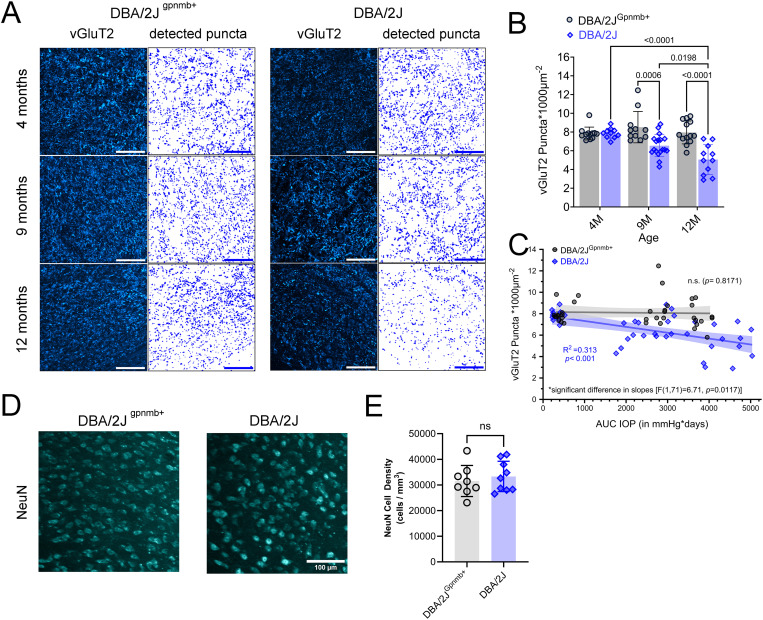
Eye pressure-associated loss of vGluT2+ puncta in the DBA/2J dLGN. **(A)** 2-photon microscopy images of vGluT2+ RGC axon terminals from dLGN core and detected puncta. vGluT2+ dLGN TC neuron somas are more apparent with decline in punctate RGC axon terminal staining in 9- and 12-month-old DBA/2J images. Scale bar = 100 μm. **(B)** Group data of mean vGluT2+ densities factored by age and strain along with the results of significant (p < 0.05) pairwise Šidák’s multiple comparison test comparisons. **(C)** Scatter plot of vGluT2+ puncta densities as a function of AUC(IOP) for each strain. Simple linear regressions were used to test associations between plotting factors and slopes from each genotype differed. Shaded area around regression line represents the 95% confidence interval. **(D)** Maximum intensity projections of dLGN sections showing neurons labeled using anti-NeuN immunolabeling from 12mo DBA/2J and DBA/2J^Gpnmb+^. **(E)** Group data of NeuN-labeled cell density analysis showing there was no detectable difference between groups (p = 0.55, unpaired t-test).

To determine the impact of OHT on RGC inputs to the dLGN throughout the progression of DBA/2J glaucoma, we stained coronal dLGN sections with an antibody against vesicular glutamate transporter 2 (vGluT2) and quantified vGluT2+ puncta within a central region of the dLGN core as a measure of RGC axon terminal density [22,24] (Fig 2A). No age-related differences were detected between the mean vGluT2+ puncta densities of DBA/2J^Gpnmb+^ control samples (4mo-9mo: *p* = 0.85; 9mo-12mo: *p* = 0.94; Šídák’s multiple comparison test) (Fig 2B). In contrast, when comparing DBA/2J vGluT2+ puncta densities across ages, we observed a lower vGluT2+ puncta density at the 12mo time point (4mo-12mo: *p* < 0.0001; 9mo-12mo: *p* = 0.020; Šídák’s multiple comparison test). Both 9- and 12mo DBA/2J samples exhibited significantly lower vGluT2+ puncta densities than age-matched DBA/2J^Gpnmb+^ controls (9mo: *p* = 0.0006; 12m: *p* < 0.0001; Šídák’s multiple comparison test) (Fig 2B). We next asked whether there was an association between OHT and vGluT2+ puncta density. We found that dLGN vGluT2+ density was negatively correlated with IOP in DBA/2J mice (R^2^ = 0.313, *p* = 0.0002). For DBA/2J^Gpnmb+^ control mice, in contrast, there was no significant correlation of vGluT2 density with IOP (*p* = 0.82). The slopes of the regressions of DBA/2J and DBA/2J^Gpnmb+^ control data were significantly different, [*F*(1,71)=6.71, *p* = 0.012], confirming strain specificity (Fig 2C).

In a subset of 12mo DBA/2J and DBA/2J^Gpnmb+^ control samples, we labeled neurons using an anti-NeuN antibody to test for glaucoma/IOP-dependent loss of post-synaptic neurons in the dLGN (Fig 2D and 2E). We have previously reported an atrophy of dLGN TC neuron somata in the DBA/2J dLGN [24], but did not detect a change in neuron density in this analysis, which was 31562 ± 6065 NeuN+ cells/mm^3^ in the DBA/2J^Gpnmb+^ (n = 8) and 33331 ± 5917 NeuN+ cells/mm^3^ in DBA/2J (n = 9; *p* = 0.55, unpaired t-test), suggesting that dLGN neurons are not lost at the oldest time point in the current study.

**Fig 3:**
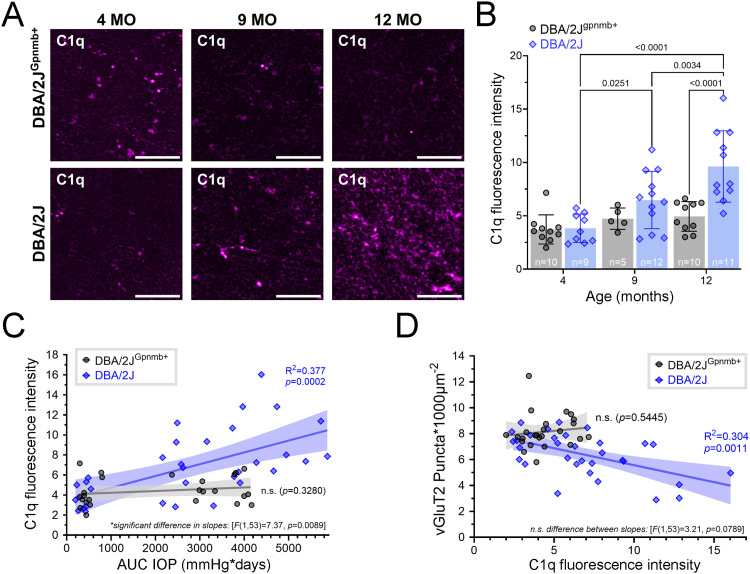
C1q intensity increases as a function of age and eye pressure in DBA/2J dLGNs. **(A)** Representative images of anti-C1q immunostaining of dLGN tissue obtained from 4-, 9-, and 12-month-old DBA/2J and DBA/2J^Gpnmb+^ mice (scale bar = 20 μm). **(B)** Bar graph factoring age and strain into peak C1q fluorescence intensities that were measured within the highest-expressing plane of C1q-stained dLGN volumetric images. Bar height and error bar represent the mean±SD. Sample size (number of mice) for each condition are located within bars. Significant (p < 0.05) pair-wise comparisons (two-tailed, Šidák-corrected multiple comparison tests) are shown with p-values. **(C)** Individual C1q intensities plotted against their corresponding IOP integrals for each strain, overlaid with line fits and 95% confidence intervals resulting from simple linear regressions and follow-up slope comparisons. **(D)** Scatterplot of C1q intensity as a function of vGluT2+ puncta density in DBA/2J and DBA/2J^Gpnmb+^ dLGNs with linear regression outputs and follow-up slope comparison results.

To investigate whether vGluT2+ loss coincides with an increase in the presence of molecular machinery related to synaptic pruning, we stained a subset of DBA/2J (n = 25) and DBA/2J^Gpnmb+^ (n = 22) dLGN tissue sections with an antibody against complement component C1q (Fig 3A), which is a molecular tag that facilitates the removal of excess retinogeniculate synapses during development [48,53,54]. Pairwise comparisons of mean C1q staining intensities revealed that C1q expression within the dLGN was low and unchanging for DBA/2J^Gpnmb+^ controls across all time points (4mo-9mo: *p* = 0.79; 9mo-12mo: *p* > 0.99; 4mo-12mo: *p* = 0.52; two-tailed Šidák-corrected multiple comparisons tests). In contrast, there was an age-related increase in C1q peak intensity in DBA/2J sections (4mo-9mo: *p* = 0.025; 9mo-12mo: *p* = 0.0034; 4mo-12mo: *p* < 0.0001; two-tailed Šidák multiple comparisons tests) that could be statistically distinguished from age-matched controls by 12 months of age (*p* < 0.0001, Šidák multiple comparisons test) (Fig 3B). Given the timing of the C1q increase observed in DBA/2J samples, we hypothesized that C1q values might be associated with OHT, so we paired C1q-peak-intensities with their corresponding IOP values and performed simple linear regressions (Fig 3C). Indeed, C1q labeling intensity significantly correlated with IOP in the dLGN of DBA/2J mice [*F*(1,30)=18.7, *p* = 0.0002], but not in DBA/2J^Gpnmb+^ controls [*F*(1,23)=0.999, *p* = 0.33]. Slope comparison showed that the DBA/2J and DBA/2J^Gpnmb+^ control datasets differed from each other [*F*(1,53)=7.37, *p* = 0.0089] (Fig 3C). We next asked whether C1q intensities corresponded to vGluT2 staining loss in the dLGN, finding a negative correlation of vGluT2+ puncta density with C1q labeling intensity for DBA/2J mice [*F*(1,30)=13.1, R^2^ = 0.304, *p* = 0.0011], whereas there was no such significant relationship for the DBA/2J^Gpnmb+^ controls (Fig 3D). Despite the difference, no distinction could be made between the regressions between strains [*F*(1,53)=3.21, *p* = 0.079] (Fig 3D).

**Fig 4:**
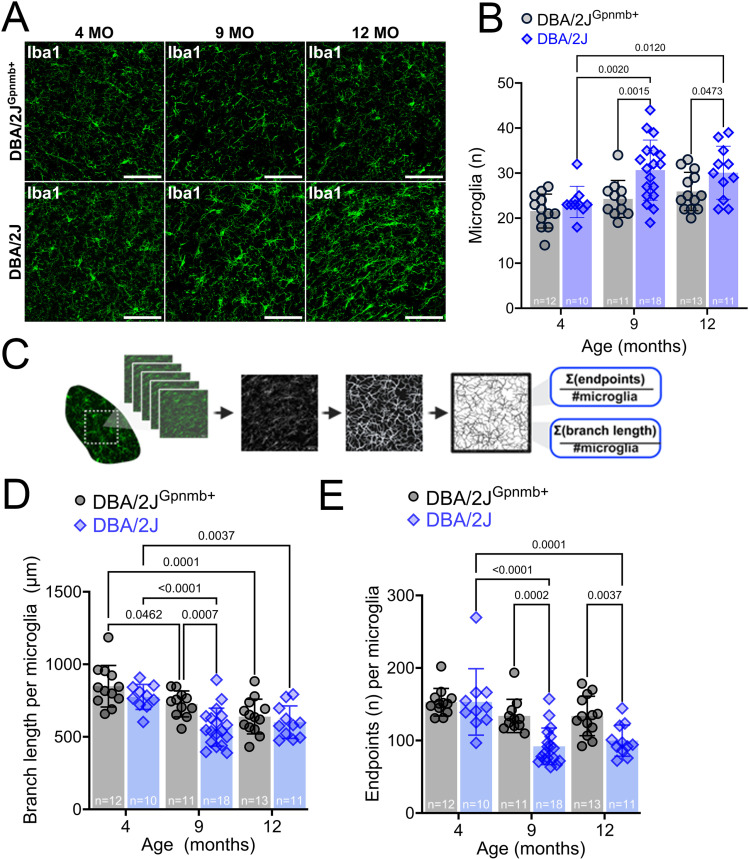
Morphology of DBA/2J dLGN-resident microglia at glaucoma-relevant time points. **(A)** Representative maximum intensity projections of 2-photon-acquired image stacks (z-depth: 40 μm) for visualizing dLGN-resident microglia (Iba1 immunostained) in 4-, 9-, and 12-month-old DBA/2J and DBA/2J^Gpnmb+^ controls. Scale bar: = 100 µm. **(B)** Group data showing the number of microglia contained within each volumetric dLGN image stack for 39 DBA/2J (blue, diamonds) and 36 DBA/2J^Gpnmb+^ (gray, circles) mice. Pairwise comparisons are p-values from Šidák-corrected multiple comparison tests. **(C)** Illustrative workflow showing skeletonization of Iba1 images and analysis of total microglia branch lengths and quantification of endpoints to investigate microglia morphology. **(D)** Group data of microglia branch length from the skeleton analysis. Pairwise comparisons are p-values from Šidák-corrected multiple comparison tests. **(E)** Group data of microglia endpoints from the skeleton analysis. Pairwise comparisons are p-values from Šidák-corrected multiple comparison tests.

We next sought to test whether IOP and/or age impacted the number or morphology of microglia in the dLGN. We predicted that high IOP would evoke a shift of microglia morphology toward a more “active” state. Therefore, we obtained DBA/2J and DBA/2J^Gpnmb+^ dLGN tissue at three pathologically relevant ages for the purpose of characterizing the microglial response to OHT in the dLGN throughout the progression of DBA/2J glaucoma. This was tested by performing a variety of morphometric analyses on Iba1+ cells within the dLGN to quantify features relevant to microglial ramification. There was a significant effect of both age [*F*(2,69)=7.996, *p* = 0.0008] and strain [*F*(1,69)=12.53, *p* = 0.0007] on Iba1+ cell counts (two-way ANOVA). Iba1+ cell counts from 4-month-old samples showed 23 ± 4 microglia in our imaging volume, or 4200 ± 730 microglia/mm^3^. Pairwise comparisons between Iba1+ cell counts in DBA/2J^Gpnmb+^ control samples revealed there was no effect of age, with Iba1+ cell counts being similar from 4 to 12 months of age [4mo-9mo: *t*(69)=1.279, *p* = 0.4980; 9mo-12mo: *t*(69)=0.7996, *p* = 0.8116; 4mo-12mo: *t*(69)=2.152, *p* = 0.1011] (Fig 4B). In the DBA/2J dLGN, microglia cell density was higher at 9mo and 12mo time points (9mo: 31 ± 7; 12mo: 30 ± 6), compared to 4mo (24 ± 3) [4mo-9mo *t*(69)=3.56, *p* = 0.0021; 4mo-12mo, *t*(69)=2.95, *p* = 0.013; (Šidák multiple comparison; Fig 4B]. Although microglia cell counts were higher in DBA/2J dLGNs at 12mo (30 ± 6) compared to 4mo DBA/2J dLGNs [*t*(69)=2.95, *p* = 0.130], they did not differ from 9mo DBA/2J counts [*t*(69)=0.2986, *p* = 0.9872 (Šidák multiple comparison; Fig 4B)].

Next, we assessed microglial ramification by quantifying microglia cell branch length and endpoint averages from analysis of skeletonized Iba1 fluorescence images [50] (Fig 4C–4F). Descriptions of arbor complexity are a powerful tool for characterizing the state of microglia polarization within a neurodegenerative context because microglia exist along a morphological continuum that ranges from intricately branched under homeostatic/surveilling conditions to ameboid during immune activation. In DBA/2J^Gpnmb+^ and DBA/2J dLGN tissue sections, there appeared to be both age- and strain-dependent effects on microglia branch length based on 2-way ANOVA results [age: *F*(2,69)=17.6, *p* < 0.0001; strain: *F*(1,69)=10.7, *p* = 0.0016]. Šidák multiple comparisons tests further revealed reduced branch length at 9mo and 12mo in both DBA/2J and DBA/2J^Gpnmb+^ samples. Comparison of branch length between 9mo DBA/2J^Gpnmb+^ and DBA/2J revealed a lower branch length in DBA/2J samples (p = 0.0007) (Fig 4D).

Analysis of the number of microglia endpoints, another measure of microglial complexity, revealed a similar pattern (Fig 4E), with a 2-way ANOVA revealing significant effects of both age [*F*(2,69)=14.88, *p* < 0.0001] and genotype [*F*(1,69)=15.05, *p* = 0.0002]. There was no significant difference between 9mo and 12mo DBA/2J^Gpnmb+^ microglia, but there was a lower number of microglia endpoints per cell in the 9mo and 12mo samples compared to 4mo. Additionally, comparisons of DBA/2J^Gpnmb+^ with DBA/2J at 9mo revealed lower numbers of microglia endpoints, again indicating a simplified morphology consistent with a move away from the surveilling/homeostatic phenotype.

**Fig 5:**
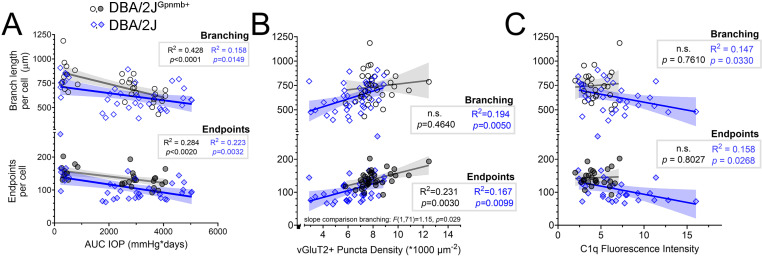
DBA/2J glaucoma is accompanied by age- and eye pressure-associated changes to microglia morphology within the visual thalamus. **(A)** Scatterplots and linear regressions of microglia branch length and endpoints per cell from skeleton analysis plotted against AUC(IOP). Shaded area represents the 95% confidence interval. Black circles: DBA/2J^Gpnmb+^ controls. Blue diamonds: DBA/2J. **(B)** Scatterplots and linear regressions, as in **A** of microglia skeleton parameters plotted against vGluT2 puncta density. **(C)** Scatterplots and linear regressions of microglia parameters plotted against C1q fluorescence intensity.

We next sought to determine whether OHT and loss of RGC axon terminals might be related to microglial ramification within the visual thalamus, since these microglia rapidly respond to changes within synaptic microenvironments. We tested this by performing linear regressions to assess potential relationships of OHT, vGluT2+ RGC axon terminal density, and complement C1q with microglia morphology within the visual thalamus (Fig 5A–5C). We observed negative associations between IOP and measures of Iba1+ cell complexity (Fig 5A); for both DBA/2J^Gpnmb+^ controls and DBA/2J mice, Iba1+ cell branch length was negatively correlated with AUC(IOP) (Fig 5A). Likewise, the number of microglia endpoints was negatively correlated with IOP for both DBA/2J^Gpnmb+^controls and DBA/2J Iba1+ cells. For both endpoints and branching, slope comparison results showed that IOP-associations were strain-independent [branching: *F*(1,64)=2.32, *p* = 0.132; endpoints: *F*(1,64)=0.264, *p* = 0.609].

We next assessed whether features of Iba1+ complexity were associated with vGluT2+ puncta density (Fig 5B). Indeed, we found a linear association between Iba1+ branch length and vGluT2+ puncta density for DBA/2J mice, but not for DBA/2J^Gpnmb+^ controls. We confirmed the relationship of branch length and vGluT2 puncta density to be independent of strain by comparing the slope outputs of strain-specific regressions, finding no significant difference in slopes from each regression [F(1,71)=1.15, p = 0.29]. Regarding microglial endpoints, we found that the number of endpoints per cell was associated with vGluT2+ puncta densities in both DBA/2J mice and DBA/2J^Gpnmb+^ controls. Additionally, there was no significant difference in slopes from each regression [F(1,71)=0.03, p = 0.86].

We found that measures of microglia complexity were significantly correlated with C1q intensity in dLGNs of DBA/2J mice but not in DBA/2J^Gpnmb+^ controls (Fig 5C); DBA/2J Iba1+ cell branching and endpoint measurements both decreased as a function of C1q intensity and there was no such relationship for DBA/2J^Gpnmb+^ controls.

**Fig 6:**
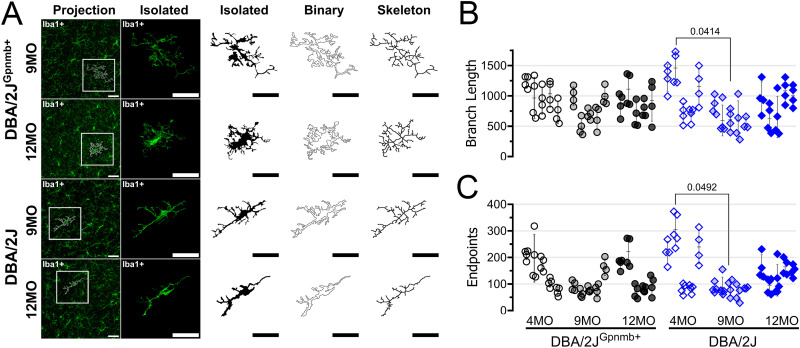
Reconstruction of individual microglia morphology. **(A)** Example images and reconstruction of randomly-selected Iba1 immunolabeled microglia from 9- and 12-month old (MO) dLGN sections from DBA/2J^Gpnmb+^ and DBA/2J mice. **(B & C)** Skeleton analysis of branch length and endpoints of four individual microglia cells per mouse. Each column of data points contains four cells from a single animal. Pairwise comparisons represent significant (p < 0.05) p-values from Šidák-corrected multiple comparison tests.

**Fig 7:**
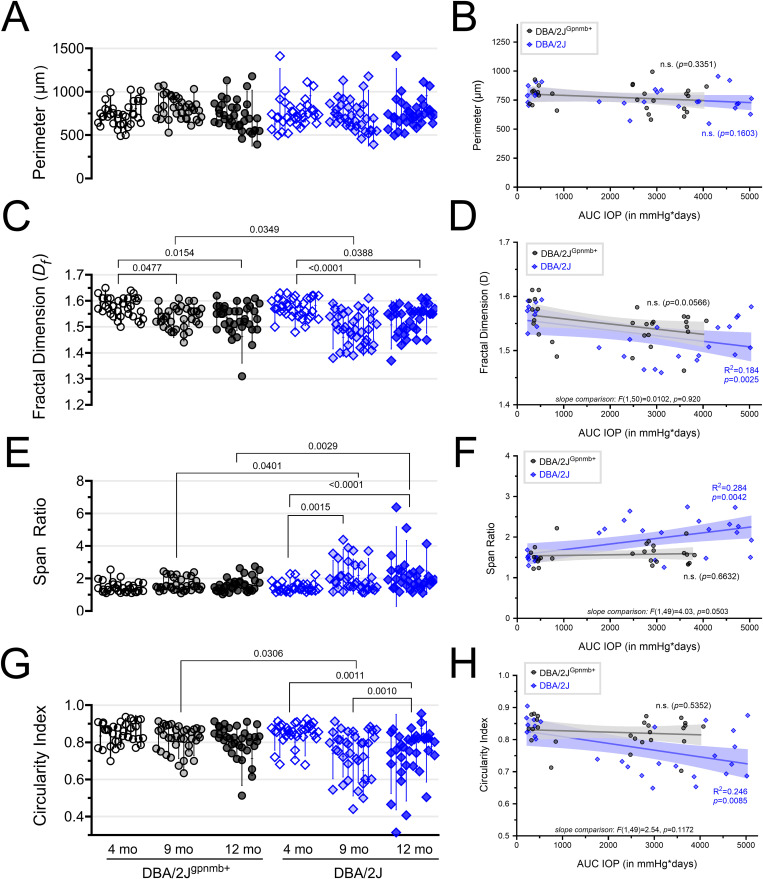
Fractal analysis of microglia morphology reveals a shift toward rod-like microglia morphology in aged DBA/2J mice. **(A)** Plot of microglia perimeter (# pixels) output of the FracLac analysis from four randomly selected and manually-reconstructed microglia cells per mouse of DBA/2J^Gpnmb+^ controls (black circles) and DBA/2J mice (blue diamonds) at 4, 9, and 12 months of age. Each column of data points corresponds to measurements from a single animal. **(B)** Scatterplot and simple linear regression of microglia perimeter measurements averaged from individual animals plotted against AUC(IOP). Shaded area represents the 95% confidence intervals. **(C & D)** Similar to **A** and **B**, plotting the FracLac parameter “Fractal Dimension” (*D*_*f*_). Pairwise comparisons represent significant (p < 0.05) results of Šidák multiple comparison tests following a nested one-way ANOVA (p < 0.0001). **(E–H)** Similar analyses as A-D for FracLac parameters “Span Ratio” and “Circularity Index”.

In addition to taking on simplified/ameboid morphologies, microglia can respond to central nervous system injury by taking on an elongated/rod/bipolar-like morphology [44–47]. These rod microglia might play important roles in phagocytosis of cellular debris and/or repair of central nervous system circuits. Qualitatively, we observed that many Iba1-labeled cells were elongated and aligned in a common direction in dLGN sections from aged DBA/2J mice, suggesting that OHT might trigger adoption of a rod-like morphology in the dLGN (Fig 4A). To quantitatively analyze microglia morphology and test this, we performed a fractal analysis of individual microglia cells. We randomly selected four individual microglia cells from each of our multiphoton image stacks and reconstructed 2-dimensional binarized projections (Fig 6A). Skeleton analysis of these individual microglia generally affirmed results from the global analysis (Fig 6B and 6C), showing a lower number of endpoints and lower total branch length in the 9mo DBA/2J compared to 4mo DBA/2J, although we did not detect significant differences when compared to age matched controls, which likely results from variability and inclusion of only four individual microglia cells per mouse. We next used the FracLac ImageJ plug-in to analyze cell perimeter, circularity, fractal dimension (*D*_*f*_), and span ratio of each cell (Fig 7). We hypothesized that microglia perimeters would decrease with simplifying their morphologies with age and in ocular hypertensive dLGN, but perimeter was indistinguishable between all conditions (Fig 7A), as revealed by a nested one-way ANOVA [*F(*5,48)=1.013, *p* = 0.4025]. Accordingly, we found that perimeter of individual microglia cells was not significantly correlated with IOP for either strain (DBA2J: *F*(1,24)=0.968, *p* = 0.335; DBA/2J^Gpnmb+^ controls: *F*(1,25)=2.09, *p* = 0.1603) (Fig 7B).

Fractal dimension (*D*_*f*_) provides a general measure of microglia process complexity, and this value revealed a pattern that mirrored measurements we obtained using skeleton analyses of process length and endpoints (Fig 4). Specifically, we found a reduction in *D*_*f*_ with increasing age in both DBA/2J^Gpmnb+^ controls and DBA/2J mice (one-way ANOVA), revealing significant differences between 9-month control and DBA/2J measurements. Likewise, *D*_*f*_ was negatively correlated with AUC(IOP) for DBA/2J mice. A similar regression analysis for the DBA/2J^Gpnmb+^ control population had a p-value of 0.057 and comparison of the slopes between genotypes showed they were similar [F(1,50)=0.01, p = 0.92]. This result, which examines microglia complexity without consideration of shape, aligns closely with our findings from the global skeleton analysis, highlighting an apparent age- and IOP-dependent reduction in microglia in both DBA/2J and DBA/2J^Gpnmb+^ controls with more pronounced effects observed in DBA/2J at the 9mo time point.

To specifically examine microglia elongation and test whether they adopt a more bipolar/rod morphology in the dLGN in response to OHT, we analyzed two parameters: 1) the *span ratio*, which is the ratio of the longest axis to the orthogonal axis of a convex hull drawn around the microglial cell, with higher values indicating a more elongated morphology, and 2) *circularity index*, which quantifies how close the convex hull is to a circle on a scale of 0–1, with 1 being circular. For *span ratio*, microglia cells from older DBA/2J tissue sections had higher values, and a one-way nested ANOVA revealed a significant difference among groups. Pairwise analyses using Šídák’s multiple comparisons tests showed that DBA/2J microglia *span ratios* were significantly higher than controls at both glaucoma-relevant time points (9mo: p = 0.0401; 12mo: p = 0.0028) and increased with age in the DBA/2J population while *span ratios* were unchanged across age for the DBA/2J^Gpnmb+^ controls. *Span ratio* was also significantly correlated with AUC(IOP) in the DBA/2J population, but not the DBA/2J^Gpnmb+^ controls, although comparisons of the slopes of the two regressions had a p-value of 0.050. Analysis of *circularity index* data revealed a similar pattern. Following a significant nested one-way ANOVA [F(5,48)=7.514, p < 0.0001], results of two-tailed Šídák-corrected t-tests showed lower *circularity index* values of cells from older DBA/2J tissue sections, revealing a loss of circularity compared to age-matched controls (p = 0.0306). An age-related loss of microglial circularity was detected by comparisons of DBA/2Js to their 9mo (p = 0.0011) and 12mo (p = 0.0010) counterparts. In a similar fashion to *span ratio*, the *circularity index* negatively correlated with AUC(IOP) in the DBA/2J population, but not the DBA/2J^Gpnmb+^ controls, although comparison of the slopes of the two regressions had a p-value of 0.12. These analyses highlight an adoption of an elongated/rod-like morphology of dLGN microglia with increasing cumulative IOP specifically in DBA/2J mice and not DBA/2J^Gpnmb+^ controls.

**Fig 8:**
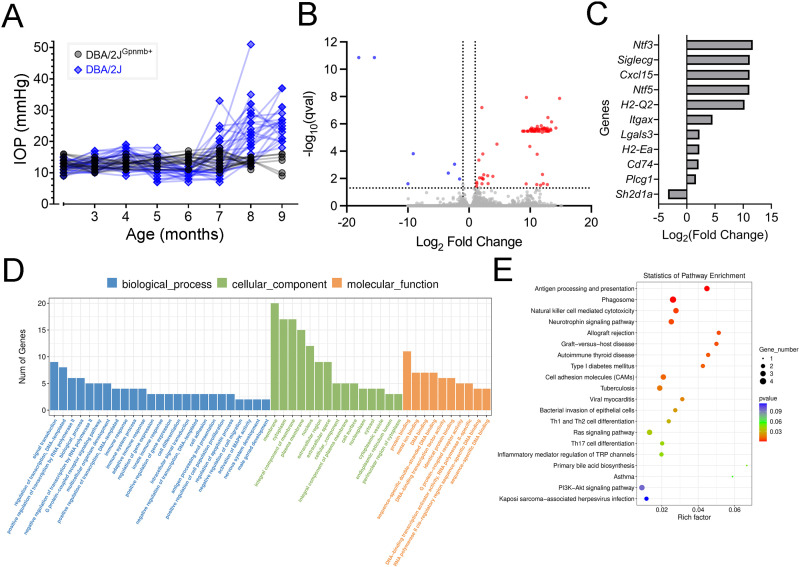
Alterations in gene expression in the dLGN of DBA/2J mice revealed by RNA sequencing. **(A)** Intraocular pressure measurements from DBA/2J^Gpnmb+^ (n = 18 eyes, 9 mice) and DBA/2J (n = 30 eyes,15 mice. **(B)** Volcano plot of 33,064 genes showing 77 that were significantly upregulated and 7 that were significantly downregulated in the 9 month-old DBA/2J dLGN relative to the age-matched controls. Significantly up- or down-regulated genes were identified by criteria of a 1.5-fold change and false discovery rate-corrected p-value < 0.05. dLGN tissue from three mice was pooled for each sample (DBA/2J: 5 samples, 15 mice; DBA/2J^Gpnmb+^: 3 samples, 9 mice) **(C)** Up- and down-regulated genes with involvement in immune- and neurotrophin-related processes. **(D)** Gene ontology enrichment analysis of differentially expressed genes. **(E)** Kyoto Encyclopedia of Genes and Genomes (KEGG) enrichment analysis.

To further test for impacts of elevated IOP, we performed bulk RNA-sequencing of microdissected dLGN tissue from 9mo DBA/2J and DBA/2J^Gpnmb+^ control mice. This time point was selected to identify gene expression changes at an intermediate phase of glaucoma progression amid elevated IOP and ongoing pathological progression. After monthly IOP measurements (Fig 8A), dLGN tissue from both right and left hemispheres from three animals per genotype was pooled to give five DBA/2J samples (total = 15 mice) and three DBA/2J^Gpnmb+^ samples (total = 9 mice). We identified a total of 77 upregulated and 7 downregulated differentially expressed genes (DEGs; Fig 8B) that met criteria for differential expression (≥1.5 fold-change, q < 0.05). Roles of these genes were identified using a gene ontology (GO) analysis and Kyoto Encyclopedia of Genes and Genomes (KEGG) enrichment analysis tools (Fig 8C–8E). Among the pathways identified in GO analysis were several related to immune system function, including “immune response” (GO:0006955), “innate immune response” (GO:0045087), and “antigen processing and presentation” (GO:0019882). Likewise, top results on KEGG analysis included “antigen processing and presentation”, “phagosome”, and “natural killer cell mediated cytotoxicity”. Upregulated protein-coding genes included major histocompatibility complex (MHC) class I and class II genes (*H2-Q2*, *Cd74,* and *H2-Ea*), and CD11c*/Itgax* of the complement pathway, consistent with upregulated immune system function in DBA/2J dLGN. *Lgals3* was upregulated 4.8-fold. Galectin-3, which it encodes, plays roles in microglia activation and inflammatory responses. Expression of two neurotrophins, neurotrophin-3 and neurotrophin-5 (*Ntf3* and *Ntf5*) along with the gene for phospholipase C gamma 1 (*Plcg1*), a neurotrophin receptor effector, was also significantly upregulated.

## Discussion

The primary objective of this study was to determine whether glaucoma’s key risk factors - aging and elevated IOP - impact microglia within the visual thalamus of DBA/2J mice. Since control IOPs remained stable over time, their AUC(IOP) linear regression outputs served as a proxy for the effects of age, which allowed us to interpret the relative contributions of each risk factor on RGC degeneration separately. Additionally, we sought to explore relationships between dLGN microglia and the loss of vGlut2 labeling of presynaptic RGC terminals [55,56]. In the absence of high eye pressure, microglial counts were consistent across DBA/2J and DBA/2J^Gpnmb+^ dLGNs regardless of age. In contrast, OHT conditions showed comparably more Iba1+ cells, indicating a limited, albeit sustained rise in microglia presence within dLGNs of ocular hypertensive mice. Importantly, this glaucoma-specific expansion involved either the accumulation of, or conversion to, rod-like phenotypes as evidenced by fractal analysis of microglia morphology. Analysis of skeletonized microglia revealed that microglial deramification in controls occurred gradually and was visible by 12 months of age in control mice, while chronic OHT in DBA/2J mice appeared to accelerate this loss of complexity in an IOP-dependent manner. Deramification of dLGN microglia during aging in controls was not accompanied by a loss of RGC terminals or changes in C1q levels, but multiple geometric features associated with rod microglia did correlate with the IOP-associated loss of vGluT2+ terminals, and with the heightened C1q labeling observed in glaucoma. Finally, RNA sequencing results revealed that chronic OHT upregulates several genes that have previously been reported in glaucoma research, in addition to multiple immune-related genes that may be relevant to the role of rod microglia in the dLGN. Overall, these data highlight the confluence of aging and OHT in altering the state of dLGN microglia during RGC degeneration in glaucoma.

### Age and OHT differentially impact microglia morphology in the visual thalamus

In healthy tissues, microglia maintain highly-branched, ramified morphologies and motile processes that allow them to actively monitor and engage with local synapses [13,54]. Results from our global skeleton analysis revealed that aging associates with a gradual loss of microglial complexity in the dLGN without overt pathological consequences. The negative correlation of microglia endpoints with vGluT2+ puncta density regardless of strain or glaucoma status might imply that reduced microglial contact in the dLGN compromises presynaptic integrity even in the absence of underlying disease, which would implicate sustained microglial deramification as a potential threat to synapses. Alternatively, this finding might reflect a proportional relationship between the degree of RGC innervation and the amount of microglial surveillance in the dLGN.

### Rod microglia in the glaucomatous dLGN

As the central nervous system’s primary sentinels of insult and injury, microglia are extremely plastic and can actively shift in and out of context-dependent states [57,58]. Here, using a fractal analysis of microglia morphology, we found that chronic IOP elevation led to a striking accumulation of Iba1+ cells in the visual thalamus that took on rod-like, bipolar states, which are defined by long thin cell bodies that lack nearly all protrusions [44,45,51,59,60]. Fractal measures that are sensitive to microglia elongation - *circularity* and *span ratio* - both correlated with AUC(IOP) in the DBA/2J dLGN, whereas there was no relationship in the control samples, suggesting that the rod morphology is related to glaucoma pathology and not age in DBA/2J mice. By visual inspection, rod microglia also appeared to be evenly distributed and were noticeably oriented in parallel with the optic tract. This suggests that rod microglia in the dLGN might make contact with traversing RGC axons. Indeed, close proximity to damaged neuronal compartments is commonly observed in published reports of rod microglia within ocular structures [ 44,51,59,60], and severe optic nerve injury has even resulted in trains of rod microglia fanning away from the optic disc along the axons of injured RGCs [61].

Multiple lines of evidence indicate that rod microglia are transcriptionally distinct, non-inflammatory, and highly proliferative [60]. Microglia that are restricted to sites of direct injury are often proposed to play a neuroprotective role [62,63]. In line with this, neuroprotection by rod microglia was recently demonstrated in a rat model of spinal cord injury [64]. The elongated morphology and association with neuronal processes suggest they might provide structural support to stressed or damaged axons and/or dendrites. Rod microglia have also been hypothesized to be involved in synaptic stripping, whereby synapses are removed from damaged neurons, possibly in an attempt at preserving neurons by reducing metabolic stress [44,45,47]. Indeed, synaptic stripping might be occurring in the glaucomatous dLGN, as IOP-dependent loss of vGlut2-labeled RGC axon terminals is followed at later time points by a loss of TC neuron dendrites [22].

### Altered gene expression in glaucomatous dLGN

Our transcriptomics data confirmed altered immune profiles in the dLGN of ocular hypertensive DBA/2J mice. GO and KEGG analyses of significantly up/down-regulated genes both revealed terms related to immune system function, antigen presentation, and phagocytosis. While we performed RNA sequencing on whole dLGN tissue samples, several upregulated genes such as *Cd74* and *H2-Ea* have established ties to microglial function. The H2-Ea protein is a major histocompatibility complex II (MHCII) component while Cd74 (cluster of differentiation 74 protein) functions to regulate intracellular MHCII trafficking and membrane organization [65]. MHCII expression allows for surface presentation of antigens for recruitment of T-cells that can impact inflammatory processes and neurodegeneration [66]. MHCII expression by microglia is relatively low under basal conditions, but increases in microglia in response to disease states, meaning its expression can serve as a marker of microglia activation [66–68]. *Itgax*, which was upregulated approximately 26-fold, encodes the complement protein, Integrin alpha-X/CD11c, that is found on a neuroprotective population of microglia [69]. CD11c+ microglia are neuroprotective in development [69] and promote a regional bias towards myelin preservation [70,71]. In the developing brain, CD11c + is associated with a seven-fold increase in galectin-3 (*Lgals3*) expression [69]. *Lgals3*, which encodes a member of beta-galactoside binding lectin family, Gal-3 [72,73], was found to be upregulated in retinal microglia in an inducible rodent glaucoma study [74] in addition to DBA/2J dLGN in the current study. Gal-3 can be membrane-bound or secreted and its function is highly context-dependent. Gal-3 that is secreted by microglia after CNS injury suppresses proinflammatory cytokines and facilitates the removal of myelin debris, which in turn promotes effective remyelination [75]. In the mature brain, Gal-3 signaling occurs between microglia and injured oligodendrocytes during the remedial response to cuprizone-induced demyelination [76].

We also found increased expression of *Ntf3* and *Ntf5*, which encode Neurotrophin-3 (NT-3) and Neurotrophin-5 (NT-5). Neurotrophin expression can be upregulated in activated microglia [77,78] and serves a neuroprotective effect through activation of TrkC and TrkB receptors [79]. Expression of *Plcg1*, which encodes phospholipase C gamma 1 (PLCγ1), was elevated by approximately 3-fold in DBA/2J dLGN. PLCγ1 is a downstream effector of Trk receptors in the brain and mediates neurotrophin effects on neuron development and plasticity [80,81]. NT-3 signaling between microglia regulates proliferation and phagocytosis [77] and can influence MHCII expression [82]. Another neurotrophin, BDNF, can act through neuronal TrkB receptors, which are also activated by NT-3 and NT-5, to influence synaptic plasticity and neuronal excitability in the dLGN [83]. Although *Bdnf* expression was not altered in the DBA/2J dLGN, it is possible that microglial NT-3 and/or NT-5 mediate TC neuron homeostatic responses to IOP [22,24,30,84–86].

### dLGN microglia and glaucoma progression in DBA/2J mice

DBA/2J mice develop elevated ocular hypertension and glaucoma due to mutations in the *Tyrp1* and *Gpnmb* genes [39,40]. In DBA/2J mice, the *Tyrp1*^*b*^ and *Gpnmb*^*R150X*^ mutations lead to iris atrophy and pigment dispersion that obstructs aqueous outflow and ultimately triggers numerous pathological signs of glaucoma in the retina and optic nerve including optic nerve cupping and degeneration, diminished optic nerve transport, RGC dendritic and synaptic remodeling, RGC somatic loss, retinal microgliosis, diminished pattern electroretinogram [38,41,87–95]. Retinal projection targets in the DBA/2J mice also show signs of glaucomatous pathology such as atrophy and loss of RGC axon terminals accompanied by functional loss of RGC output synapses [22,24,26,31]. Neurons in the dLGN that are post-synaptic to RGCs are also affected, displaying somatic atrophy and dendritic pruning, altered intrinsic excitability, and plasticity of inhibitory GABA_A_ receptors [22,24,30]. Many of these measures also show correlations with IOP in DBA/2J mice, pointing to a link between eye pressure and effects. Thus, although there is no established link between *Gpnmb* or *Tyrp1* mutations and human glaucoma [96,97] and the DBA/2J model has some potential shortcomings for certain applications [98,99], the OHT and glaucoma phenotype in DBA/2J mice still makes it useful for studying the impact of IOP on visual system structure and function.

This extensive literature provides a solid foundation for staging numerous aspects of glaucomatous pathology in DBA/2J mice for comparison with the effects on dLGN microglia in the present study. We examined dLGN microglia at 4, 9, and 12 months of age, which represent snapshots of three distinct phases of glaucoma progression in these mice (Fig 1). In summary, 4 month DBA/2J mice largely resembled their DBA/2J^Gpnmb+^ control counterparts in all parameters we measured. This is unsurprising, as this is prior to IOP elevation and most prior studies have revealed few if any signs of pathology at this time point, with exception of some microglia activation in the inner retina [91] and modestly impaired anterograde axon transport in some DBA/2J mice [26]. By 9 months, we detected a modest loss of RGC axon terminals, an increase in C1q labeling, and some simplifying and elongation of microglia morphology in DBA/2J mouse dLGN. This corresponds to detectable decline of RGC synaptic output along with detectable dLGN relay neuron hyperexcitability and somatic atrophy from our prior work [22,24]. PERG amplitudes are clearly reduced in an IOP-dependent manner at this time point, which is consistent with loss of RGC axons and glial scarring in the optic nerve [87,88,92]. There is evidence for altered RGC gene expression at this stage along with increased apoptosis and diminished numbers of RBPMS-immunopositive RGCs [24,94,100]. This likely points to RGC somatic degeneration, although other work using alternative labeling methods has suggested that RGC somas persist despite the apparent damage to RGC axons distal to the optic nerve head [22,100]. At this time point, we found a clear divergence of dLGN microglia morphology between DBA/2J and DBA/2J^Gpnmb+^ controls, with DBA/2J microglia showing morphological simplification and adoption of a rod-like phenotype. At 12 months of age, rod microglia morphology is still apparent in the DBA/2J dLGN. At this point, these mice show signs of severe IOP-related optic nerve damage including reduced axon density and increased glial scarring with dramatically reduced PERG amplitudes and RGC dendritic pruning and synapse loss [87–90,92,93]. Within the superior colliculus, RGC axon terminals persist, but show signs of atrophy and mitochondrial abnormalities [31]. In the dLGN, there was no detectable loss of NeuN+ neurons at this time point, although we have previously documented some neuronal somatic atrophy at an earlier time point [24] and loss of dLGN relay neuron dendritic complexity at 12 months [22]. However, vGlut2 loss is more pronounced at 12 months than at 9 months, and this is accompanied by a nearly 60% reduction in the strength of RGC synaptic input to dLGN TC neurons [22,24].

Results of the current study contribute additional data to the documented timeline of glaucoma progression in DBA/2J mice. While retinal synaptic rearrangement, optic nerve pathology, and anterograde transport deficits are relatively early events [6,7], altered dLGN microglia morphology largely matches the timeline for altered dLGN function [22]. In this case, microglia simplification and adoption of rod morphology appears to correspond in time to diminished retinogeniculate synaptic strength and loss of vGlut2-labeled RGC axon terminals. This suggests that microglia are responding to OHT-triggered pathological stress to RGC axons and synaptic outputs in the dLGN.

### Limitations and future directions

The current study focused on IOP-related alterations to microglia morphology in the dLGN of DBA/2J mice. While morphology is clearly related to microglia function, we do not have a clear picture of the precise role microglia play in the glaucomatous dLGN. Future experiments depleting microglia from mice with glaucoma could help determine whether they ultimately play a supportive/neuroprotective role or whether microglia activation is a net detriment in the glaucomatous dLGN. Such experiments are bound to be complicated, as a recent preprint showed disassembly of synapses in the retina following experimental IOP elevation was reduced by a microglial inhibitor [101], but 10-week-long systemic depletion of microglia in DBA/2J mice led to more severe optic nerve damage [102], suggesting that microglia might have divergent effects depending on disease stage or RGC compartment. Future studies using inducible models such as the microbead occlusion model [103] or silicone oil ocular hypertension approach [104] will be informative, and some evidence for microglia activation and even rod-like morphology is apparent in the dLGN from a study using the microbead approach [74]. Although we did not investigate whether rod-like microglia are dLGN-resident microglia that have transitioned morphologies or if they are a population that has infiltrated, the stability of microglia numbers from 9- to 12-months suggests that they are resident microglia, as recruited macrophages are usually transient. Elsewhere, increases in rod microglia were reported to occur via local proliferation [60,61] rather than peripheral recruitment, but this would need to be tested under these conditions. While RNAseq analysis was conducted in tissue from 9mo DBA/2J and DBA/2J^Gpnmb+^ controls, in an effort to capture a relatively early time point in the disease process, a picture of differential gene expression at a later time point with more pronounced glaucoma pathology might reveal a different transcriptomic profile. Additionally, this relied on analysis of whole tissue isolates and pooling of tissue from several mice. This can introduce noise into the gene expression data, possibly hiding altered gene expression in specific dLGN cell types and masking the biological variability arising from the diverse IOP profiles in individual mice. Future work using either single-cell sequencing [15] or sequencing of microglia-enriched samples could help address this and allow for a clearer picture of microglia-specific IOP-related expression changes and functional implications.

## Supporting information

S1 Dataset Supporting data for Figs 1–7. Microsoft Excel spreadsheet containing values from IOP measurements, vGluT2 density measurements, measures of microglia number and process complexity, fractal analysis of microglia, and C1q analysis.
(XLSX)

S2 Dataset RNA-sequencing dataset for Fig 8. Microsoft Excel spreadsheet containing a list of genes and associated analysis (gene description, gene ontology, KEGG, fold change, q-values) from bulk RNA sequencing of dLGN tissue from DBA/2J and DBA/2JGpnmb+ mice.
(XLSX)

## References

[1] Flaxman SR, Bourne RRA, Resnikoff S, Ackland P, Braithwaite T, Cicinelli MV, et al. Global causes of blindness and distance vision impairment 1990-2020: a systematic review and meta-analysis. Lancet Glob Health. 2017;5(12):e1221–34. doi: 10.1016/S2214-109X(17)30393-5 29032195

[2] Lommatzsch C, van Oterendorp C. Current Status and Future Perspectives of Optic Nerve Imaging in Glaucoma. J Clin Med. 2024;13(7):1966. doi: 10.3390/jcm13071966 38610731 PMC11012267

[3] Quigley HA, Broman AT. The number of people with glaucoma worldwide in 2010 and 2020. Br J Ophthalmol. 2006;90(3):262–7. doi: 10.1136/bjo.2005.081224 16488940 PMC1856963

[4] Tham Y-C, Li X, Wong TY, Quigley HA, Aung T, Cheng C-Y. Global prevalence of glaucoma and projections of glaucoma burden through 2040: a systematic review and meta-analysis. Ophthalmology. 2014;121(11):2081–90. doi: 10.1016/j.ophtha.2014.05.013 24974815

[5] Weinreb RN, Aung T, Medeiros FA. The pathophysiology and treatment of glaucoma: a review. JAMA. 2014;311(18):1901–11. doi: 10.1001/jama.2014.3192 24825645 PMC4523637

[6] Calkins DJ. Critical pathogenic events underlying progression of neurodegeneration in glaucoma. Prog Retin Eye Res. 2012;31:702–19. doi: 10.1016/j.preteyeres.2012.07.001 22871543 PMC3472111

[7] Calkins DJ. Adaptive responses to neurodegenerative stress in glaucoma. Prog Retin Eye Res. 2021;84:100953. doi: 10.1016/j.preteyeres.2021.100953 33640464 PMC8384979

[8] Seabrook TA, El-Danaf RN, Krahe TE, Fox MA, Guido W. Retinal input regulates the timing of corticogeniculate innervation. J Neurosci. 2013;33(24):10085–97. doi: 10.1523/JNEUROSCI.5271-12.2013 23761904 PMC3682386

[9] Morrison JC, Johnson EC, Cepurna W, Jia L. Understanding mechanisms of pressure-induced optic nerve damage. Prog Retin Eye Res. 2005;24(2):217–40. doi: 10.1016/j.preteyeres.2004.08.003 15610974

[10] Howell GR, Libby RT, Jakobs TC, Smith RS, Phalan FC, Barter JW, et al. Axons of retinal ganglion cells are insulted in the optic nerve early in DBA/2J glaucoma. J Cell Biol. 2007;179(7):1523–37. doi: 10.1083/jcb.200706181 18158332 PMC2373494

[11] Soto I, Howell GR. The complex role of neuroinflammation in glaucoma. Cold Spring Harb Perspect Med. 2014;4(8):a017269. doi: 10.1101/cshperspect.a017269 24993677 PMC4109578

[12] Williams PA, Marsh-Armstrong N, Howell GR, Lasker/IRRF Initiative on Astrocytes and Glaucomatous Neurodegeneration Participants. Neuroinflammation in glaucoma: A new opportunity. Exp Eye Res. 2017;157:20–7. doi: 10.1016/j.exer.2017.02.014 28242160 PMC5497582

[13] Schafer DP, Stevens B, Bennett ML, Bennett FC. Role of Microglia in Central Nervous System Development and Plasticity. Cold Spring Harb Perspect Biol. 2024;a041810. doi: 10.1101/cshperspect.a041810 39349311 PMC12487714

[14] Gao C, Jiang J, Tan Y, Chen S. Microglia in neurodegenerative diseases: mechanism and potential therapeutic targets. Signal Transduct Target Ther. 2023;8(1):359. doi: 10.1038/s41392-023-01588-0 37735487 PMC10514343

[15] Hammond TR, Robinton D, Stevens B. Microglia and the Brain: Complementary Partners in Development and Disease. Annu Rev Cell Dev Biol. 2018;34:523–44. doi: 10.1146/annurev-cellbio-100616-060509 30089221

[16] Guo L, Choi S, Bikkannavar P, Cordeiro MF. Microglia: Key Players in Retinal Ageing and Neurodegeneration. Front Cell Neurosci. 2022;16:804782. doi: 10.3389/fncel.2022.804782 35370560 PMC8968040

[17] Ma W, Wong WT. Aging Changes in Retinal Microglia and their Relevance to Age-related Retinal Disease. Adv Exp Med Biol. 2016;854:73–8. doi: 10.1007/978-3-319-17121-0_11 26427396 PMC4696750

[18] Liddelow SA, Guttenplan KA, Clarke LE, Bennett FC, Bohlen CJ, Schirmer L, et al. Neurotoxic reactive astrocytes are induced by activated microglia. Nature. 2017;541(7638):481–7. doi: 10.1038/nature21029 28099414 PMC5404890

[19] Guttenplan KA, Stafford BK, El-Danaf RN, Adler DI, Münch AE, Weigel MK, et al. Neurotoxic Reactive Astrocytes Drive Neuronal Death after Retinal Injury. Cell Rep. 2020;31(12):107776. doi: 10.1016/j.celrep.2020.107776 32579912 PMC8091906

[20] Liu Y, Wang A, Chen C, Zhang Q, Shen Q, Zhang D, et al. Microglial cGAS-STING signaling underlies glaucoma pathogenesis. Proc Natl Acad Sci U S A. 2024;121(36):e2409493121. doi: 10.1073/pnas.2409493121 39190350 PMC11388346

[21] Salkar A, Wall RV, Basavarajappa D, Chitranshi N, Parilla GE, Mirzaei M, et al. Glial Cell Activation and Immune Responses in Glaucoma: A Systematic Review of Human Postmortem Studies of the Retina and Optic Nerve. Aging Dis. 2024;15(5):2069–83. doi: 10.14336/AD.2024.0103 38502591 PMC11346413

[22] Smith JC, Zhang KY, Sladek A, Thompson J, Bierlein ER, Bhandari A, et al. Loss of Retinogeniculate Synaptic Function in the DBA/2J Mouse Model of Glaucoma. eNeuro. 2022;9(6):ENEURO.0421-22.2022. doi: 10.1523/ENEURO.0421-22.2022 36526366 PMC9794376

[23] Van Hook MJ. Influences of Glaucoma on the Structure and Function of Synapses in the Visual System. Antioxid Redox Signal. 2022;37(10–12):842–61. doi: 10.1089/ars.2021.0253 35044228 PMC9587776

[24] Van Hook MJ, Monaco C, Bierlein ER, Smith JC. Neuronal and Synaptic Plasticity in the Visual Thalamus in Mouse Models of Glaucoma. Front Cell Neurosci. 2021;14:626056. doi: 10.3389/fncel.2020.626056 33584206 PMC7873902

[25] Crish SD, Calkins DJ. Central visual pathways in glaucoma: evidence for distal mechanisms of neuronal self-repair. J Neuroophthalmol. 2015;35 Suppl 1:S29-37. doi: 10.1097/WNO.0000000000000291 26274834

[26] Crish SD, Sappington RM, Inman DM, Horner PJ, Calkins DJ. Distal axonopathy with structural persistence in glaucomatous neurodegeneration. Proc Natl Acad Sci U S A. 2010;107(11):5196–201. doi: 10.1073/pnas.0913141107 20194762 PMC2841892

[27] Bhandari A, Smith JC, Zhang Y, Jensen AA, Reid L, Goeser T, et al. Early-Stage Ocular Hypertension Alters Retinal Ganglion Cell Synaptic Transmission in the Visual Thalamus. Front Cell Neurosci. 2019;13:426. doi: 10.3389/fncel.2019.00426 31607867 PMC6761307

[28] Chen H, Zhao Y, Liu M, Feng L, Puyang Z, Yi J, et al. Progressive degeneration of retinal and superior collicular functions in mice with sustained ocular hypertension. Invest Ophthalmol Vis Sci. 2015;56(3):1971–84. doi: 10.1167/iovs.14-15691 25722210 PMC4365983

[29] Seabrook TA, Burbridge TJ, Crair MC, Huberman AD. Architecture, Function, and Assembly of the Mouse Visual System. Annu Rev Neurosci. 2017;40:499–538. doi: 10.1146/annurev-neuro-071714-033842 28772103

[30] Van Hook MJ, McCool S. Enhanced Synaptic Inhibition in the Dorsolateral Geniculate Nucleus in a Mouse Model of Glaucoma. eNeuro. 2024;11(7):ENEURO.0263-24.2024. doi: 10.1523/ENEURO.0263-24.2024 38937109 PMC11242868

[31] Smith MA, Xia CZ, Dengler-Crish CM, Fening KM, Inman DM, Schofield BR, et al. Persistence of intact retinal ganglion cell terminals after axonal transport loss in the DBA/2J mouse model of glaucoma. J Comp Neurol. 2016;524(17):3503–17. doi: 10.1002/cne.24012 27072596 PMC5050057

[32] Nuzzi R, Dallorto L, Rolle T. Changes of Visual Pathway and Brain Connectivity in Glaucoma: A Systematic Review. Front Neurosci. 2018;12:363. doi: 10.3389/fnins.2018.00363 29896087 PMC5986964

[33] Yucel YH, Gupta N. A framework to explore the visual brain in glaucoma with lessons from models and man. Exp Eye Res. 2015;141:171–8. doi: 10.1016/j.exer.2015.07.004 26169795

[34] Gupta N, Yücel YH. Brain changes in glaucoma. Eur J Ophthalmol. 2003;13 Suppl 3:S32-5. doi: 10.1177/112067210301303s06 12749675

[35] Gupta N, Yücel YH. Glaucoma and the brain. J Glaucoma. 2001;10(5 Suppl 1):S28-9. doi: 10.1097/00061198-200110001-00011 11890268

[36] Gupta N, Greenberg G, de Tilly LN, Gray B, Polemidiotis M, Yücel YH. Atrophy of the lateral geniculate nucleus in human glaucoma detected by magnetic resonance imaging. Br J Ophthalmol. 2009;93(1):56–60. doi: 10.1136/bjo.2008.138172 18697810 PMC2605243

[37] Gupta N, Ang L-C, Noël de Tilly L, Bidaisee L, Yücel YH. Human glaucoma and neural degeneration in intracranial optic nerve, lateral geniculate nucleus, and visual cortex. Br J Ophthalmol. 2006;90(6):674–8. doi: 10.1136/bjo.2005.086769 16464969 PMC1860237

[38] Libby RT, Anderson MG, Pang I-H, Robinson ZH, Savinova OV, Cosma IM, et al. Inherited glaucoma in DBA/2J mice: pertinent disease features for studying the neurodegeneration. Vis Neurosci. 2005;22(5):637–48. doi: 10.1017/S0952523805225130 16332275

[39] Howell GR, Libby RT, Marchant JK, Wilson LA, Cosma IM, Smith RS, et al. Absence of glaucoma in DBA/2J mice homozygous for wild-type versions of Gpnmb and Tyrp1. BMC Genet. 2007;8:45. doi: 10.1186/1471-2156-8-45 17608931 PMC1937007

[40] Anderson MG, Smith RS, Hawes NL, Zabaleta A, Chang B, Wiggs JL, et al. Mutations in genes encoding melanosomal proteins cause pigmentary glaucoma in DBA/2J mice. Nat Genet. 2002;30(1):81–5. doi: 10.1038/ng794 11743578

[41] John SW, Smith RS, Savinova OV, Hawes NL, Chang B, Turnbull D, et al. Essential iris atrophy, pigment dispersion, and glaucoma in DBA/2J mice. Invest Ophthalmol Vis Sci. 1998;39(6):951–62. 9579474

[42] Kleesattel D, Crish SD, Inman DM. Decreased Energy Capacity and Increased Autophagic Activity in Optic Nerve Axons With Defective Anterograde Transport. Invest Ophthalmol Vis Sci. 2015;56(13):8215–27. doi: 10.1167/iovs.15-17885 26720474 PMC5110237

[43] Dengler-Crish CM, Smith MA, Inman DM, Wilson GN, Young JW, Crish SD. Anterograde transport blockade precedes deficits in retrograde transport in the visual projection of the DBA/2J mouse model of glaucoma. Front Neurosci. 2014;8:290. doi: 10.3389/fnins.2014.00290 25278826 PMC4166356

[44] Taylor SE, Morganti-Kossmann C, Lifshitz J, Ziebell JM. Rod microglia: a morphological definition. PLoS One. 2014;9(5):e97096. doi: 10.1371/journal.pone.0097096 24830807 PMC4022629

[45] Holloway OG, Canty AJ, King AE, Ziebell JM. Rod microglia and their role in neurological diseases. Semin Cell Dev Biol. 2019;94:96–103. doi: 10.1016/j.semcdb.2019.02.005 30826549

[46] Au NPB, Ma CHE. Recent Advances in the Study of Bipolar/Rod-Shaped Microglia and their Roles in Neurodegeneration. Front Aging Neurosci. 2017;9:128. doi: 10.3389/fnagi.2017.00128 28522972 PMC5415568

[47] Giordano KR, Denman CR, Dubisch PS, Akhter M, Lifshitz J. An update on the rod microglia variant in experimental and clinical brain injury and disease. Brain Commun. 2021;3(1):fcaa227. doi: 10.1093/braincomms/fcaa227 33501429 PMC7811762

[48] Stephan AH, Barres BA, Stevens B. The complement system: an unexpected role in synaptic pruning during development and disease. Annu Rev Neurosci. 2012;35:369–89. doi: 10.1146/annurev-neuro-061010-113810 22715882

[49] Rosen AM, Stevens B. The role of the classical complement cascade in synapse loss during development and glaucoma. Adv Exp Med Biol. 2010;703:75–93. doi: 10.1007/978-1-4419-5635-4_6 20711708

[50] Young K, Morrison H. Quantifying Microglia Morphology from Photomicrographs of Immunohistochemistry Prepared Tissue Using ImageJ. J Vis Exp. 2018;(136):57648. doi: 10.3791/57648 29939190 PMC6103256

[51] Morrison H, Young K, Qureshi M, Rowe RK, Lifshitz J. Quantitative microglia analyses reveal diverse morphologic responses in the rat cortex after diffuse brain injury. Sci Rep. 2017;7(1):13211. doi: 10.1038/s41598-017-13581-z 29038483 PMC5643511

[52] Karperien A, Ahammer H, Jelinek HF. Quantitating the subtleties of microglial morphology with fractal analysis. Front Cell Neurosci. 2013;7:3. doi: 10.3389/fncel.2013.00003 23386810 PMC3558688

[53] Stevens B, Allen NJ, Vazquez LE, Howell GR, Christopherson KS, Nouri N, et al. The classical complement cascade mediates CNS synapse elimination. Cell. 2007;131(6):1164–78. doi: 10.1016/j.cell.2007.10.036 18083105

[54] Schafer DP, Lehrman EK, Kautzman AG, Koyama R, Mardinly AR, Yamasaki R, et al. Microglia sculpt postnatal neural circuits in an activity and complement-dependent manner. Neuron. 2012;74(4):691–705. doi: 10.1016/j.neuron.2012.03.026 22632727 PMC3528177

[55] Hammer S, Carrillo GL, Govindaiah G, Monavarfeshani A, Bircher JS, Su J, et al. Nuclei-specific differences in nerve terminal distribution, morphology, and development in mouse visual thalamus. Neural Dev. 2014;9:16. doi: 10.1186/1749-8104-9-16 PMC4108237 25011644

[56] Koch SM, Dela Cruz CG, Hnasko TS, Edwards RH, Huberman AD, Ullian EM. Pathway-specific genetic attenuation of glutamate release alters select features of competition-based visual circuit refinement. Neuron. 2011;71(2):235–42. doi: 10.1016/j.neuron.2011.05.045 21791283 PMC3375067

[57] Reddaway J, Richardson PE, Bevan RJ, Stoneman J, Palombo M. Microglial morphometric analysis: so many options, so little consistency. Front Neuroinform. 2023;17:1211188. doi: 10.3389/fninf.2023.1211188 37637472 PMC10448193

[58] Paolicelli RC, Sierra A, Stevens B, Tremblay M-E, Aguzzi A, Ajami B, et al. Microglia states and nomenclature: A field at its crossroads. Neuron. 2022;110(21):3458–83. doi: 10.1016/j.neuron.2022.10.020 36327895 PMC9999291

[59] Ziebell JM, Taylor SE, Cao T, Harrison JL, Lifshitz J. Rod microglia: elongation, alignment, and coupling to form trains across the somatosensory cortex after experimental diffuse brain injury. J Neuroinflammation. 2012;9:247. doi: 10.1186/1742-2094-9-247 23111107 PMC3526458

[60] Tam WY, Ma CHE. Bipolar/rod-shaped microglia are proliferating microglia with distinct M1/M2 phenotypes. Sci Rep. 2014;4:7279. doi: 10.1038/srep07279 25452009 PMC4250916

[61] Yuan T-F, Liang Y-X, Peng B, Lin B, So K-F. Local proliferation is the main source of rod microglia after optic nerve transection. Sci Rep. 2015;5:10788. doi: 10.1038/srep10788 26035780 PMC4649910

[62] Rojas B, Gallego BI, Ramírez AI, Salazar JJ, de Hoz R, Valiente-Soriano FJ, et al. Microglia in mouse retina contralateral to experimental glaucoma exhibit multiple signs of activation in all retinal layers. J Neuroinflammation. 2014;11:133. doi: 10.1186/1742-2094-11-133 25064005 PMC4128533

[63] de Hoz R, Gallego BI, Ramírez AI, Rojas B, Salazar JJ, Valiente-Soriano FJ, et al. Rod-like microglia are restricted to eyes with laser-induced ocular hypertension but absent from the microglial changes in the contralateral untreated eye. PLoS One. 2013;8(12):e83733. doi: 10.1371/journal.pone.0083733 24367610 PMC3867486

[64] Jaffer H, Andrabi SS, Petro M, Kuang Y, Steinmetz MP, Labhasetwar V. Catalytic antioxidant nanoparticles mitigate secondary injury progression and promote functional recovery in spinal cord injury model. J Control Release. 2023;364:109–23. doi: 10.1016/j.jconrel.2023.10.028 37866402 PMC10842504

[65] Potru PS, Spittau B. CD74: a prospective marker for reactive microglia? Neural Regen Res. 2023;18(12):2673–4. doi: 10.4103/1673-5374.371350 37449617 PMC10358643

[66] Schetters STT, Gomez-Nicola D, Garcia-Vallejo JJ, Van Kooyk Y. Neuroinflammation: Microglia and T Cells Get Ready to Tango. Front Immunol. 2018;8:1905. doi: 10.3389/fimmu.2017.01905 29422891 PMC5788906

[67] Wyss-Coray T, Mucke L. Inflammation in neurodegenerative disease--a double-edged sword. Neuron. 2002;35(3):419–32. doi: 10.1016/s0896-6273(02)00794-8 12165466

[68] Neumann H, Boucraut J, Hahnel C, Misgeld T, Wekerle H. Neuronal control of MHC class II inducibility in rat astrocytes and microglia. Eur J Neurosci. 1996;8(12):2582–90. doi: 10.1111/j.1460-9568.1996.tb01552.x 8996807

[69] Wlodarczyk A, Holtman IR, Krueger M, Yogev N, Bruttger J, Khorooshi R, et al. A novel microglial subset plays a key role in myelinogenesis in developing brain. EMBO J. 2017;36(22):3292–308. doi: 10.15252/embj.201696056 28963396 PMC5686552

[70] Jia J, Zheng L, Ye L, Chen J, Shu S, Xu S, et al. CD11c+ microglia promote white matter repair after ischemic stroke. Cell Death Dis. 2023;14(2):156. doi: 10.1038/s41419-023-05689-0 36828819 PMC9958101

[71] Benmamar-Badel A, Owens T, Wlodarczyk A. Protective Microglial Subset in Development, Aging, and Disease: Lessons From Transcriptomic Studies. Front Immunol. 2020;11:430. doi: 10.3389/fimmu.2020.00430 32318054 PMC7147523

[72] Yang R-Y, Rabinovich GA, Liu F-T. Galectins: structure, function and therapeutic potential. Expert Rev Mol Med. 2008;10:e17. doi: 10.1017/S1462399408000719 18549522

[73] Thomas L, Pasquini LA. Galectin-3-Mediated Glial Crosstalk Drives Oligodendrocyte Differentiation and (Re)myelination. Front Cell Neurosci. 2018;12:297. doi: 10.3389/fncel.2018.00297 30258354 PMC6143789

[74] Tribble JR, Kokkali E, Otmani A, Plastino F, Lardner E, Vohra R, et al. When Is a Control Not a Control? Reactive Microglia Occur Throughout the Control Contralateral Pathway of Retinal Ganglion Cell Projections in Experimental Glaucoma. Transl Vis Sci Technol. 2021;10(1):22. doi: 10.1167/tvst.10.1.22 33510961 PMC7804521

[75] García-Revilla J, Boza-Serrano A, Espinosa-Oliva AM, Soto MS, Deierborg T, Ruiz R, et al. Galectin-3, a rising star in modulating microglia activation under conditions of neurodegeneration. Cell Death Dis. 2022;13(7):628. doi: 10.1038/s41419-022-05058-3 35859075 PMC9300700

[76] Hoyos HC, Rinaldi M, Mendez-Huergo SP, Marder M, Rabinovich GA, Pasquini JM, et al. Galectin-3 controls the response of microglial cells to limit cuprizone-induced demyelination. Neurobiol Dis. 2014;62:441–55. doi: 10.1016/j.nbd.2013.10.023 24184798

[77] Elkabes S, DiCicco-Bloom EM, Black IB. Brain microglia/macrophages express neurotrophins that selectively regulate microglial proliferation and function. J Neurosci. 1996;16(8):2508–21. doi: 10.1523/JNEUROSCI.16-08-02508.1996 8786427 PMC6578768

[78] Elkabes S, Peng L, Black IB. Lipopolysaccharide differentially regulates microglial trk receptor and neurotrophin expression. J Neurosci Res. 1998;54(1):117–22. doi: 10.1002/(sici)1097-4547(19981001)54:1<117::aid-jnr12>3.0.co;2-4 9778155

[79] Tabakman R, Lecht S, Sephanova S, Arien-Zakay H, Lazarovici P. Interactions between the cells of the immune and nervous system: neurotrophins as neuroprotection mediators in CNS injury. Prog Brain Res. 2004;146:387–401. doi: 10.1016/s0079-6123(03)46024-x 14699975

[80] Segal RA. Selectivity in neurotrophin signaling: theme and variations. Annu Rev Neurosci. 2003;26:299–330. doi: 10.1146/annurev.neuro.26.041002.131421 12598680

[81] Huang EJ, Reichardt LF. Trk receptors: roles in neuronal signal transduction. Annu Rev Biochem. 2003;72:609–42. doi: 10.1146/annurev.biochem.72.121801.161629 12676795

[82] Neumann H, Misgeld T, Matsumuro K, Wekerle H. Neurotrophins inhibit major histocompatibility class II inducibility of microglia: involvement of the p75 neurotrophin receptor. Proc Natl Acad Sci U S A. 1998;95(10):5779–84. doi: 10.1073/pnas.95.10.5779 9576961 PMC20456

[83] Van Hook MJ. Brain-derived neurotrophic factor is a regulator of synaptic transmission in the adult visual thalamus. J Neurophysiol. 2022;128(5):1267–77. doi: 10.1152/jn.00540.2021 36224192 PMC9662800

[84] Lessmann V. Neurotrophin-dependent modulation of glutamatergic synaptic transmission in the mammalian CNS. Gen Pharmacol. 1998;31(5):667–74. doi: 10.1016/s0306-3623(98)00190-6 9809461

[85] Lessmann V, Heumann R. Modulation of unitary glutamatergic synapses by neurotrophin-4/5 or brain-derived neurotrophic factor in hippocampal microcultures: presynaptic enhancement depends on pre-established paired-pulse facilitation. Neuroscience. 1998;86(2):399–413. doi: 10.1016/s0306-4522(98)00035-9 9881855

[86] Hernández-Echeagaray E. Neurotrophin-3 modulates synaptic transmission. Vitam Horm. 2020;114:71–89. doi: 10.1016/bs.vh.2020.04.008 32723551

[87] Porciatti V. The mouse pattern electroretinogram. Doc Ophthalmol. 2007;115(3):145–53. doi: 10.1007/s10633-007-9059-8 17522779 PMC2773675

[88] Saleh M, Nagaraju M, Porciatti V. Longitudinal evaluation of retinal ganglion cell function and IOP in the DBA/2J mouse model of glaucoma. Invest Ophthalmol Vis Sci. 2007;48(10):4564–72. doi: 10.1167/iovs.07-0483 17898279 PMC2765717

[89] Berry RH, Qu J, John SWM, Howell GR, Jakobs TC. Synapse Loss and Dendrite Remodeling in a Mouse Model of Glaucoma. PLoS One. 2015;10(12):e0144341. doi: 10.1371/journal.pone.0144341 26637126 PMC4670161

[90] Williams PA, Howell GR, Barbay JM, Braine CE, Sousa GL, John SWM, et al. Retinal ganglion cell dendritic atrophy in DBA/2J glaucoma. PLoS One. 2013;8(8):e72282. doi: 10.1371/journal.pone.0072282 23977271 PMC3747092

[91] Bosco A, Steele MR, Vetter ML. Early microglia activation in a mouse model of chronic glaucoma. J Comp Neurol. 2011;519(4):599–620. doi: 10.1002/cne.22516 21246546 PMC4169989

[92] Bosco A, Breen KT, Anderson SR, Steele MR, Calkins DJ, Vetter ML. Glial coverage in the optic nerve expands in proportion to optic axon loss in chronic mouse glaucoma. Exp Eye Res. 2016;150:34–43. doi: 10.1016/j.exer.2016.01.014 26851485 PMC4972706

[93] Cooper ML, Crish SD, Inman DM, Horner PJ, Calkins DJ. Early astrocyte redistribution in the optic nerve precedes axonopathy in the DBA/2J mouse model of glaucoma. Exp Eye Res. 2016;150:22–33. doi: 10.1016/j.exer.2015.11.016 26646560 PMC4889569

[94] Schuettauf F, Rejdak R, Walski M, Frontczak-Baniewicz M, Voelker M, Blatsios G, et al. Retinal neurodegeneration in the DBA/2J mouse-a model for ocular hypertension. Acta Neuropathol. 2004;107(4):352–8. doi: 10.1007/s00401-003-0816-9 14745571

[95] Jakobs TC, Libby RT, Ben Y, John SWM, Masland RH. Retinal ganglion cell degeneration is topological but not cell type specific in DBA/2J mice. J Cell Biol. 2005;171(2):313–25. doi: 10.1083/jcb.200506099 16247030 PMC2171185

[96] Lynch S, Yanagi G, DelBono E, Wiggs JL. DNA sequence variants in the tyrosinase-related protein 1 (TYRP1) gene are not associated with human pigmentary glaucoma. Mol Vis. 2002;8:127–9. 12011806

[97] Lascaratos G, Shah A, Garway-Heath DF. The genetics of pigment dispersion syndrome and pigmentary glaucoma. Surv Ophthalmol. 2013;58(2):164–75. doi: 10.1016/j.survophthal.2012.08.002 23218808

[98] Turner AJ, Vander Wall R, Gupta V, Klistorner A, Graham SL. DBA/2J mouse model for experimental glaucoma: pitfalls and problems. Clin Exp Ophthalmol. 2017;45(9):911–22. doi: 10.1111/ceo.12992 28516453

[99] Agarwal R, Agarwal P, Iezhitsa I. Exploring the current use of animal models in glaucoma drug discovery: where are we in 2023?. Expert Opin Drug Discov. 2023;18(11):1287–300. doi: 10.1080/17460441.2023.2246892 37608634

[100] Buckingham BP, Inman DM, Lambert W, Oglesby E, Calkins DJ, Steele MR, et al. Progressive ganglion cell degeneration precedes neuronal loss in a mouse model of glaucoma. J Neurosci. 2008;28(11):2735–44. doi: 10.1523/JNEUROSCI.4443-07.2008 18337403 PMC6670674

[101] Yu A, Tan LX, Lakkaraju A, Santina LD, Ou Y. Microglia target synaptic sites early during excitatory circuit disassembly in neurodegeneration. bioRxiv. 2024:2024.06.13.598914. doi: 10.1101/2024.06.13.598914 40212592 PMC11984620

[102] Diemler CA, MacLean M, Heuer SE, Hewes AA, Marola OJ, Libby RT, et al. Microglia depletion leads to increased susceptibility to ocular hypertension-dependent glaucoma. Front Aging Neurosci. 2024;16:1396443. doi: 10.3389/fnagi.2024.1396443 39015474 PMC11250491

[103] Sappington RM, Carlson BJ, Crish SD, Calkins DJ. The microbead occlusion model: a paradigm for induced ocular hypertension in rats and mice. Invest Ophthalmol Vis Sci. 2010;51(1):207–16. doi: 10.1167/iovs.09-3947 19850836 PMC2869054

[104] Zhang J, Li L, Huang H, Fang F, Webber HC, Zhuang P, et al. Silicone oil-induced ocular hypertension and glaucomatous neurodegeneration in mouse. Elife. 2019;8:e45881. doi: 10.7554/eLife.45881 31090540 PMC6533060

